# Supplementary material for: Effects of online group exercises for older adults on physical, psychological and social wellbeing: a randomized pilot trial
Source: PeerJ. 2017 Apr 5;5:e3150. doi: 10.7717/peerj.3150 (PMC5384569; doi:10.7717/peerj.3150)
Supplement: Table S1 [file peerj-05-3150-s001.docx]

| EXERCISES | PROGRESSION LEVELS | | | | | | | | | |
| --- | --- | --- | --- | --- | --- | --- | --- | --- | --- | --- |
|  | 1 | 2 | 3 | 4 | 5 | 6 | 7 | 8 | 9 | 10 |
| Standing;  eyes closed | 1x 10s | 2x 10s | 2x 10s | 3x 10s | 3x 10s | 3x 10s  (1x US) | 3x 10s  (1x US) | 3x 10s  (2x US) | 3x 10s  US | 3x 10s  US |
| Back extension | 1x | 2x | 3x | 3x | 5x | 5x | 8x | 8x | 10x | 10x |
| Turn trunk  Left-Right | 3L;  3R | 5L;  5R | 8L;  8R | 10L;  10R | 2x 8L;  2x 8R | 2x 8L;  2x 8R | 2x 10L;  2x 10R | 2x 10L;  2x 10R | 3x 10L;  3x 10R | 3x 10L;  3x 10R |
| Foot in front | 2x 10s | 2x 10s | 2x 15s | 2x 15s | 2x 20s | 2x 20s | 2x 25s | 2x 25s | 2x 30s | 2x 30s |
| Knee bends | 1x | 2x | 3x | 5x | 5x  (1x US) | 8x  (1x US) | 8x  (2x US) | 10x  (2x US) | 10x  (3x US) | 10x  (3x US) |
| Sideways stepping (stp)  5 steps L; 5 steps R | 1 stp L;  1 stp R | 2 stp L;  2 stp R | 3 stp L;  3 stp R | 3 stp L;  3 stp R | 4 stp L;  4 stp R | 1x | 2x | 3x | 5x | 5x |
| Heel stand (hs)  Level 4>: heel walk(hw) | 8hs | 10hs | 2x 8hs | 1hw | 5hw | 8hw | 10hw | 2x 8hw | 2x 10hw | 3x 8hw |
| Toe stand (ts)  Level 4>: toe walk(tw) | 8ts | 10ts | 2x 10ts | 1tw | 5tw | 8tw | 10tw | 2x 8tw | 2x 10tw | 3x 8tw |
| Standing;  leg front-back | 5L; 5R | 8L; 8R | 10L; 10R | 10L; 10R * | 10L; 10R * | 15L; 15R * | 15L; 15R * | 20L; 20R * | 20L; 20R * | 25L; 25R * |
| Side hip | 5L; 5R | 8L; 8R | 10L; 10R | 10L; 10R * | 10L; 10R * | 15L; 15R * | 15L; 15R * | 20L; 20R * | 20L; 20R * | 25L; 25R * |
| Sitting; stretch legs independently | 8L;  8R | 10L;  10R | 2x 8L;  2x 8R | 2x 10L;  2x 10R | 2x 10L;  2x 10R * | 2x 10L;  2x 10R * | 3x 10L;  3x 10R * | 2x 10L;  2x 10R hold | 3x 10L;  3x 10R hold * | 3x 10L;  3x 10R  hold * |
| Standing on 1 leg | 8L;  8R | 2x 8L;  2x 8R | 2x 10L; 2x 10R | 2x 10L; 2x 10R * | 2x 10L; 2x 10R * | 3x 10L; 3x 10R * | 2x 10L; 2x 10R  hold * | 3x 10L; 3x 10R hold * | 3x 10L; 3x 10R hold * | 3x 10L;  3x 10R  hold * |
| Standing on 1 leg (moving) | 8L; 8R | 2x8L; 2x8R | 2x 10L; 2x 10R | 2x 10L; 2x 10R * | 2x 10L; 2x 10R * | 3x 10L; 3x 10R * | 2x 10L; 2x 10R hold * | 3x 10L; 3x 10R hold * | 3x 10L; 3x 10R hold * | 3x 10L;  3x 10R  hold * |

| L = Left leg or to the left | N = Normal speed | US = Unsupported | ( ) = Optional | * = Using ankle weights |
| --- | --- | --- | --- | --- |
| R = Right leg or to the right | F = Fast speed | NH = No hands | hold = Sustain position | x = Repetitions |
