# Supplementary figures and images for: Effects of online group exercises for older adults on physical, psychological and social wellbeing: a randomized pilot trial
Source: PeerJ. 2017 Apr 5;5:e3150. doi: 10.7717/peerj.3150 (PMC5384569; doi:10.7717/peerj.3150)

# Social interactions in the social group

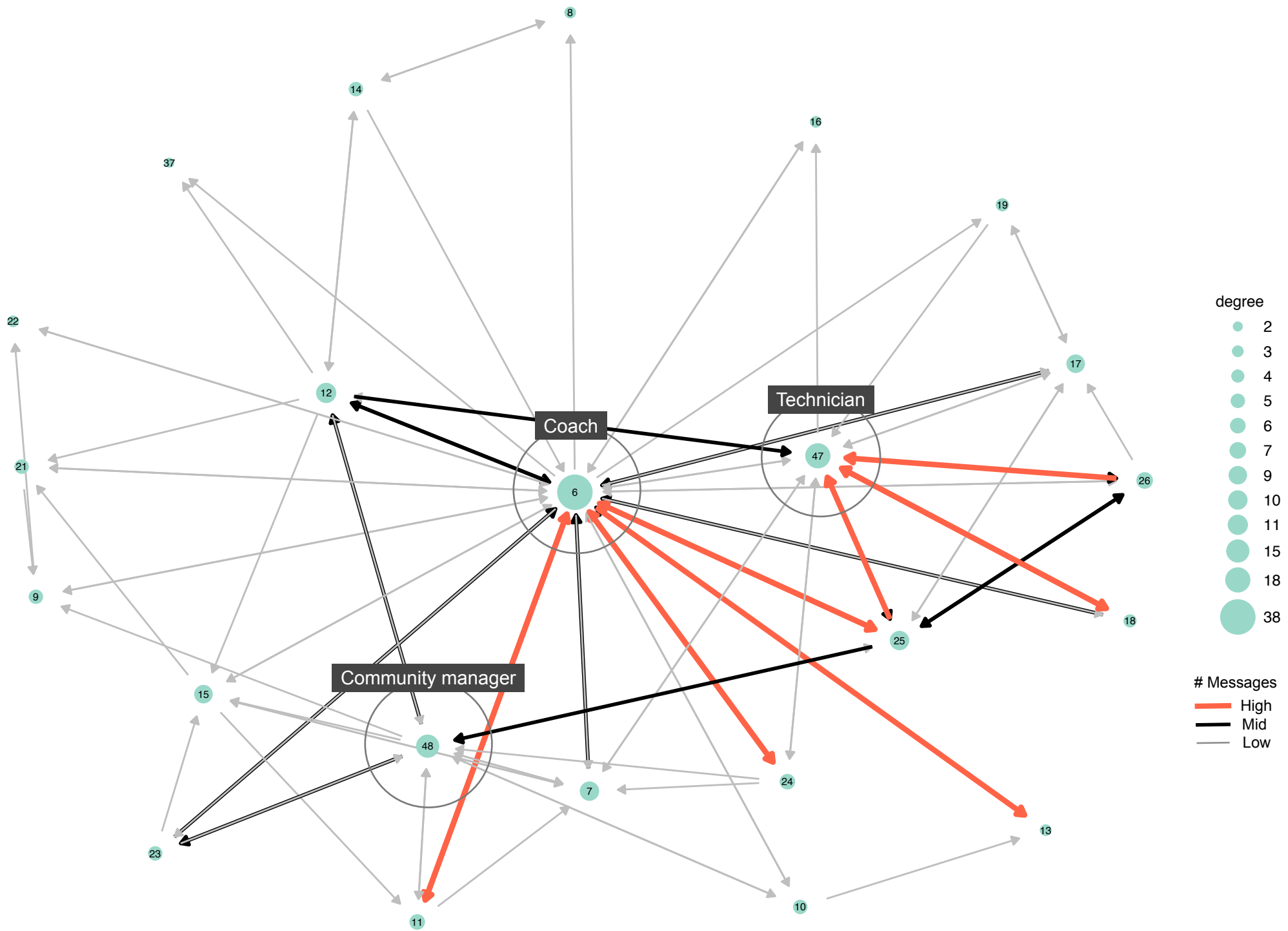

Supplement: Figure S1 [file peerj-05-3150-s002.pdf]
